# Exploring Career Prospects in Oral and Maxillofacial Pathology and Oral Medicine Among Final‐Year Brazilian Dental Students: A Multi‐institutional Study

**DOI:** 10.1111/jop.70152

**Published:** 2026-05-13

**Authors:** Hélen Kaline Farias Bezerra, Fabrício Emanuel Soares de Oliveira, Daniella Reis Barbosa Martelli, Renato Assis Machado, Zêus Araújo Cunha, Fábio Ramoa Pires, Danyele Cambraia Franco de Souza, Silvia Regina de Almeida Reis, Giovanna Ribeiro Souto, Paulo Rogério Ferreti Bonan, Danyel Elias da Cruz Perez, Alan Roger dos Santos‐Silva, Luiz Evaristo Ricci Volpato, Janete Dias Almeida, Hercílio Martelli‐Júnior

**Affiliations:** ^1^ Oral Diagnosis Department, Piracicaba Dental School University of Campinas Piracicaba São Paulo Brazil; ^2^ Montes Claros State University, Health Sciences Postgraduate Program Montes Claros Minas Gerais Brazil; ^3^ São Judas Tadeu University São Paulo São Paulo Brazil; ^4^ State University of Rio de Janeiro and Estácio de Sá University Rio de Janeiro Rio de Janeiro Brazil; ^5^ José Do Rosário Vellano University (Centrinho), Institute of Dentistry and Health Sciences Alfenas Minas Gerais Brazil; ^6^ Department of Basic Science Bahiana School of Medicine and Public Health Salvador Bahia Brazil; ^7^ School of Dentistry, Pontifical Catholic University of Minas Gerais (PUC Minas) Belo Horizonte Brazil; ^8^ Federal University of Paraíba, School of Dentistry João Pessoa Paraíba Brazil; ^9^ Department of Clinical and Preventive Dentistry Universidade Federal de Pernambuco Recife Pernambuco Brazil; ^10^ School of Dentistry, University of Cuiabá Cuiabá Mato Grosso Brazil; ^11^ Universidade Estadual Paulista (UNESP), Instituto de Ciência e Tecnologia, Câmpus São José dos Campos São Paulo Brazil; ^12^ Oral Pathology and Oral Medicine, Dental School, Montes Claros State University Montes Claros Minas Gerais Brazil

**Keywords:** career choice, dental, oral medicine, pathology oral, students

## Abstract

**Background:**

Oral and maxillofacial pathology (OP) and oral medicine (OM) are recognized specialties in Brazil and worldwide. Identifying dental students' intentions to pursue these fields and understanding the factors influencing their career choices may provide insights into the future of the specialties. Therefore, this study aimed to determine the intention of Brazilian final‐year dental students to pursue the professional career in OP and OM.

**Methods:**

Final‐year undergraduate dental students, from various public and private institutions in Brazil, were invited to voluntarily complete a self‐administered and anonymous virtual questionnaire. The questionnaire consisted of 11 questions. The statistical analysis consisted of describing the absolute and relative numbers, and performing analytical statistics using Pearson's chi‐squared test for categorical variables.

**Results:**

A total of 358 students participated of the study. The majority were female (75.1%), with a mean age of 25.27 years, mainly from public universities (52%). The main careers pursued were restorative dentistry (39.1%), dental implantology (37.7%), prosthodontics (33%), and orofacial harmonization (31.3%). The main reasons for choosing specialization were to stand out in the profession and to perform better in the profession with better clinical outcomes. OM was reported as a career choice by 17% of students and OP by 7.5%. The motivations for such choice were vocation and influence from professors, colleagues, or family members.

**Conclusion:**

The results revealed a surprising and concerning finding: interest in the fields of OP and OM, which are fundamental for diagnosing and treating complex conditions of the oral cavity, was relatively low.

## 1 Introduction

According to the Brazilian Federal Council of Dentistry (CFO), there are 131 831 dentists with registered specializations currently. Of these, 1050 (0.8%) are certified stomatologists/oral medicine clinicians and 426 (0.3%) are oral and maxillofacial pathologists [1]. In Brazil, oral and maxillofacial pathology (OP) and stomatology/oral medicine (OM) are professional specialties offered through postgraduate programs in dentistry. OP was recognized as a specialty by the CFO in 1971, while OM is a more recent specialty, recognized in 1992 [2]. These specialties are inseparable and collaborate in the diagnosis and management of oral and salivary glands diseases, temporomandibular disorders, orofacial pain, oral complications of systemic diseases, and care of systemically complex patients [1, 3].

Although no national or international guidelines currently define the “ideal” number of specialists in OP and OM, several studies have highlighted a significant shortage of professionals in these fields. A recent survey in 11 Latin American countries revealed that all of them had an insufficient number of specialists relative to their populations; in Brazil, for example, there are only 422 OP and 1072 OM specialists for over 200 million inhabitants [4]. On a global scale, the median number of oral pathologists has been estimated at just 0.48 per million inhabitants, with substantial variability in training models across countries [5]. Furthermore, in several regions, such as Europe and the Middle East, OM is still not officially recognized as a specialty, which restricts training opportunities and further contributes to the scarcity of professionals [6, 7].

For a professional, to become an oral pathologist or stomatologist in Brazil, there are two options—completing a *Lato sensu* specialization course (usually 2 years) and requesting to the CFO for the title of specialist (https://website.cfo.org.br/) or to complete a master's and/or PhD program (*Stricto sensu*) in these fields. *Lato sensu* postgraduate programs are more focused on clinical/histopathological practice, while *Stricto sensu* courses are more focused on training in teaching and research, although they also include clinical/histopathological training [1].

Considering the importance of career definition on life, studying the plans of dental students allows for an understanding of the psychological factors involved, future professional satisfaction, and the assessment of expectations related to this decision [8]. Moreover, identifying dental students who are motivated to choose these specialties as their future careers may provide valuable information on the general perception of these specialties among students, allowing measurements of students' attraction for these areas, in addition to understanding the development of OP and OM as specialties in Brazil. We highlight that a study evaluating the number of specialist dentists in the areas of OP and OM in 11 Latin American countries found an insufficient number of professionals relative to the populations in all the countries evaluated [4].

Although some studies have been conducted to understand career choices in dentistry in general [9, 10, 11, 12] or even specific areas [8, 13], there are no previous studies that aimed to comprehensively investigate students' career intentions in OP and OM. Recognizing the documented global trend of lower popularity of pediatric dentistry (OP) and orthodontics (OM) as career choices, this study adopted an exploratory, descriptive design. Its primary aims were (1) to map the academic profile and postgraduation career intentions of final‐year dental students in Brazil and, within this broader context and (2) to specifically identify the actual level of interest and the primary motivations for pursuing a career in OP and OM.

## 2 Materials and Methods

This was a cross‐sectional study on a convenience sample of final‐year undergraduate dental students from 12 Brazilian dental schools (six public and six private institutions), who voluntarily agreed to participate in this study by giving informed consent. The study was approved by the institutional research ethics committee on May 18, 2023 (protocol number # 68413223.5.0000.5418). Convenience sampling was used to try to encompass a larger number of students, inviting all students from the final year of the participating institutions. This approach was chosen due to the exploratory nature of the research and practical constraints in accessing a randomized national sample.

Participating institutions were from the Brazilian states of Bahia, Minas Gerais, Mato Grosso, Paraíba, Pernambuco, Rio de Janeiro, and São Paulo. Public dental schools were Piracicaba Dental School (FOP‐UNICAMP), Federal University of Pernambuco (UFPE), State University of Montes Claros (UNIMONTES), State University of Rio de Janeiro (UERJ), State University of São Paulo (UNESP) campus São José dos Campos, and Federal University of Paraíba (UFPB). The private institutions comprised Pontifical Catholic University of Minas Gerais (PUC‐Minas), University of Cuiabá (UNIC), Estácio de Sá University (UNESA), São Judas Tadeu University (USJT), Professor Edson Antônio Velano University (UNIFENAS), and Bahiana School of Medicine and Public Health (EBMSP).

A structured questionnaire was designed and sent to a researcher of the study at each of the 12 dental schools. Each researcher was responsible for administering the questionnaire at their respective institution. The questionnaire was administered during periods that did not interfere with students' school activities. The survey instrument was a virtual, self‐administered, and anonymous questionnaire hosted online (Google Forms), containing 11 multiple‐choice questions. A preliminary pilot study was carried out in order to test the feasibility of the questionnaire. The questionnaire comprised questions about sociodemographic information of the students, aspects of the institution where they were enrolled in, the existence of a previous higher degree, the students' plans for postgraduation training and motivations to choose a professional pathway. All specialties recognized by the CFO in Brazil were included in the questionnaire. Dental students were gathered face‐to‐face and invited to voluntarily complete the virtual questionnaire on their mobile devices.

The collected data were tabulated in Excel spreadsheets (Microsoft, Redmond, USA) and analyzed using Statistical Package for the Social Sciences (SPSS) software version 27.0 (IBM Inc., Chicago, IL, USA). Descriptive statistics were used to analyze the distribution of absolute and relative numbers, with their respective confidence intervals (95% CI), for sociodemographic characteristics and chosen specialization areas. We performed Pearson's chi‐square tests with Bonferroni correction for multiple comparisons to compare the chosen areas and reasons for choices, as well as to compare choices between students from public and private universities. A *p* ≤ 0.05 was considered as an indication of statistical significance.

We evaluated the representativeness of our convenience sample by comparing key demographic characteristics (age, sex, and institution type) against national reference data for 2023 dental graduates from the 2023 National Education Census data from the Brazilian National Institute of Educational Studies and Research (INEP). Age distributions were compared using independent samples *t*‐tests, while sex and institution type (public/private) distributions were assessed using Pearson's chi‐square tests with Yates' continuity correction. The reference data were obtained from the official INEP interactive dashboard, accessible at: https://app.powerbi.com/view?r=eyJrIjoiMGJiMmNiNTAtOTY1OC00ZjUzLTg2OGUtMjAzYzNiYTA5YjliIiwidCI6IjI2ZjczODk3LWM4YWMtNGIxZS05NzhmLWVhNGMwNzc0MzRiZiJ9&pageName=ReportSection4036c90b8a27b5f58f54.

## 3 Results

A total of 358 final‐year dental students participated in this study. While the convenience sampling method limits the generalizability of the findings, it should be noted that the achieved sample represents a substantial proportion of the target population, corresponding to 61.3% of all final‐year students (*n* = 584) enrolled in the participating dental schools during the study period.

Most of the participants were females (*n* = 269; 75.1%) and 89 (24.9%) were males. The mean age was 25.27 years (standard deviation = 5.43; age range 20–56 years). A majority of the dental students were single (*n* = 308; 86%). Considering Brazilian states, most of the sample were from Minas Gerais (*n* = 131; 36.6%), followed by Bahia (*n* = 57; 15.9%), São Paulo (*n* = 49; 13.7%), Mato Grosso (*n* = 37; 10.3%), Rio de Janeiro (*n* = 32; 8.9%), Paraíba (*n* = 29; 8.1%), and Pernambuco (*n* = 23; 6.4%). Detailed students' characterization is described in Table 1.

**TABLE 1:** Final‐year dental students' characteristics (*n* = 358).

Variables	*n* (%)	95% CI
Age	25.27 (5.43)^a^	—
Sex		
Female	269 (75.1)	70.4–79.3
Male	89 (24.9)	20.6–42.1
University		
Public	186 (52)	46.7–57.1
Private	172 (48)	42.9–53.2
Brazilian state		
Bahia	57 (15.9)	12.5–20.1
Minas Gerais	131 (36.6)	31.7–41.7
Mato Grosso	37 (10.3)	75.9–13.9
Paraíba	29 (8.1)	5.7–11.4
Pernambuco	23 (6.4)	4.3–9.4
Rio de Janeiro	32 (8.9)	6.4–12.3
São Paulo	49 (13.7)	10.5–17.6
Marital status		
Married	41 (11.5)	8.5–15.1
Divorced/separated	8 (2.2)	1.1–4.3
Single	308 (86)	82.1–89.2
Widowed	1 (0.3)	0.1–1.6
Previous degree		
Yes	62 (17.3)	13.7–21.6
No	296 (82.7)	78.4–86.2
If postgraduation is not immediate to graduation, they are planning to start postgraduation in:		
1 year	80 (22.3)	18.3–26.9
2 years	16 (4.5)	2.7–7.1
3 years	4 (1.1)	0.4–2.8
Never	1 (0.3)	0.1–1.6

^a^ Mean (standard deviation).

Most students (*n* = 186%–52%) were enrolled in public dental schools, and 172 (48%) were from private institutions. Dentistry was the first higher degree pursued by 82.7% of the students (*n* = 296), but 17.3% (*n* = 62) had already completed other courses. As the sample consisted of final‐year students, 276 (77%) indicated that they were interested in starting a postgraduate program immediately after graduating, while 82 (22.9%) were not concerned about this. Many students plan to start a postgraduate program within a year of graduation (*n* = 80; 22.3%) (Table 1).

**FIGURE 1:**
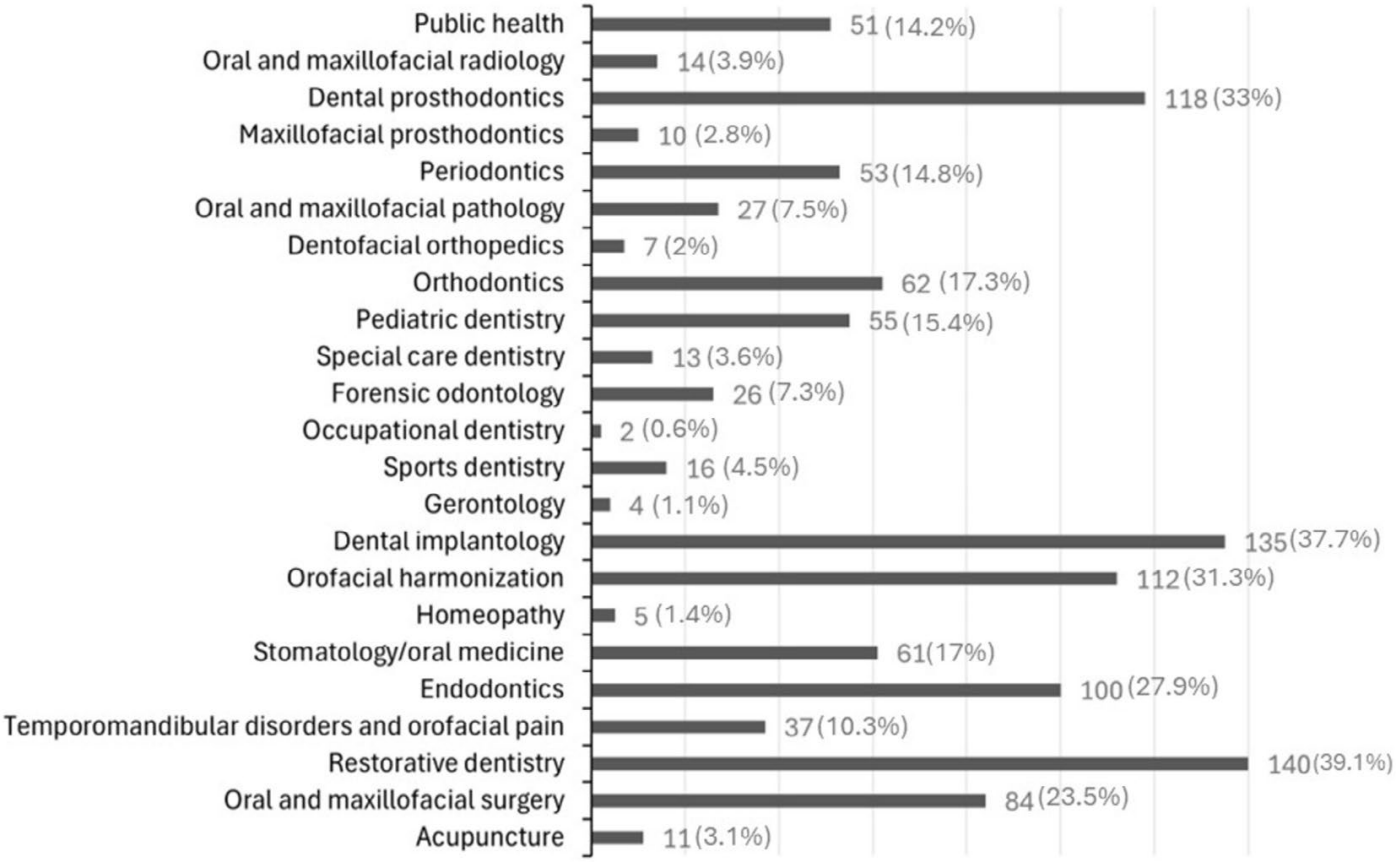
Fields of interest reported by final‐year dental students.

Considering the professional prospects of final‐year dental students, most of them were particularly interested in working in restorative dentistry (*n* = 140; 39.1%), dental implantology (*n* = 135; 37.7%), prosthodontics (*n* = 118; 33%), and orofacial harmonization (*n* = 112; 31.3%). Regarding OM, 61 (17%) of the students were interested in this professional career, and 27 (7.5%) are interested in the field of OP. Students were allowed to choose more than one answer in this question (Figure 1).

Table 2 shows the main motivations reported by the students for pursuing a career in the four main specialties of interest in this study. For all of them (restorative dentistry, orofacial harmonization, dental implantology, and prosthodontics), the main reasons for choosing specialization were to stand out in the profession and to perform better in the profession with better clinical outcomes.

**TABLE 2:** Final‐year dental students' motivations for choosing the four preferred specialties (*n* = 358).

Motivations	Restorative dentistry	Orofacial harmonization	Dental implantology	Dental prosthodontics
	*n* (%)*p* ^a^	*n* (%)*p* ^a^	*n* (%)*p* ^a^	*n* (%)*p* ^a^
Quicker financial return	65 (46.4) 1.000	52 (46.4) 1.000	71 (52.6) **0.020**	51 (43.2) 1.000
Professional distinction	92 (65.7) 1.000	71 (63.4) 1.000	93 (68.9) 1.000	84 (71.2) 0.357
Increased chance of promotion	18 (12.9) 1.000	12 (10.7) 1.000	16 (11.9) 1.000	15 (12.7) 1.000
Exploring new fields	29 (20.7) 1.000	23 (20.5) 1.000	33 (24.4) 1.000	28 (23.7) 1.000
Strengthening the professional curriculum	67 (47.9) 1.000	46 (41.1) 1.000	72 (53.3) 0.170	57 (48.3) 1.000
Networking	30 (21.4) 1.000	28 (25.0) 0.469	31 (23) 1.000	23 (19.5) 1.000
Becoming an entrepreneur	40 (28.6) 1.000	43 (38.4) **< 0.001**	48 (35.6) **< 0.001**	29 (24.6) 1.000
Improving clinical treatment results	100 (71.4) **< 0.001**	70 (62.5) 1.000	78 (57.8) 1.000	78 (66.1) 0.928

^a^ Pearson's chi‐square test with Bonferroni adjustment. Bolded values represent significance.

On the other hand, for OM, influence from a professor, family member, or colleague (*n* = 36; 59%), and vocation (*n* = 25; 43.1%) were the main motivations to pursue this career. Interestingly, for OP, vocation (*n* = 15; 60%) and influence from a professor, family member, or colleague (*n* = 13; 48.1%) were also cited as the main reasons for choosing this career (Table 3).

**TABLE 3:** Final‐year dental students' motivations for choosing oral medicine and oral pathology (*n* = 358).

Motivations	Oral medicine	Oral pathology
	*n* (%)*p* ^a^	*n* (%)*p* ^a^
Vocation	25 (43.1) 1.000	15 (60) 1.000
Interest and job opportunities	18 (29.5) **< 0.001**	6 (22.2) 0.087
Interest in other related areas which can enhance the career	24 (39.3) **< 0.001**	10 (37) **< 0.001**
Previous academic experience in these fields (internship/mentoring/research training)	23 (37.7) **< 0.001**	10 (37) **< 0.001**
Influence from a teacher, family, or colleague	36 (59) **< 0.001**	13 (48.1) **< 0.001**

^a^ Pearson's chi‐square test with Bonferroni adjustment. Bolded values represent significance.

Table 4 presents the interest in specialization stratified by public and private universities. Overall, students from public universities showed greater interest in specializing, with a notable difference in interest in Public Health (20.4% public university vs. 7.6% private university). Regarding the objective of this study, our results show a low interest of dentists in the fields of OM and OP, which implies a lack of specialists.

**TABLE 4:** Comparison of specialization areas in dentistry chosen by final‐year students from public and private institutions (*n* = 358).^a^

Area of specialization	Public university*n* (%)	Private university*n* (%)	*p*
Acupuncture	7 (3.8)	4 (2.3)	1.000
Oral and maxillofacial surgery and traumatology	46 (24.7)	38 (22.1)	1.000
Restorative dentistry	75 (40.3)	65 (37.8)	1.000
Temporomandibular dysfunction and orofacial pain	23 (12.4)	14 (8.1)	1.000
Endodontics	59 (31.7)	41 (23.8)	0.097
Oral medicine	37 (19.9)	24 (14)	1.000
Homeopathy	2 (1.1)	3 (1.7)	1.000
Orofacial harmonization	61 (32.8)	51 (29.7)	1.000
Implantology	66 (35.5)	69 (40.1)	1.000
Geriatric dentistry	2 (1.1)	2 (1.2)	1.000
Sports dentistry	9 (4.8)	7 (4.1)	1.000
Occupational dentistry	1 (0.5)	1 (0.6)	1.000
Forensic dentistry	15 (8.1)	11 (6.4)	1.000
Dentistry for patients with special needs	6 (3.2)	7 (4.1)	1.000
Pediatric dentistry	28 (15.1)	27 (15.7)	1.000
Orthodontics	29 (15.6)	33 (19.2)	1.000
Functional jaw orthopedics	7 (3.8)	0 (0)	0.660
Oral pathology	14 (7.5)	13 (7.6)	1.000
Periodontics	29 (15.6)	24 (14)	1.000
Oral and maxillofacial prosthetics	4 (2.2)	6 (3.5)	1.000
Dental prosthetics	61 (32.8)	57 (33.1)	1.000
Dental radiology and imaging	10 (5.4)	4 (2.3)	1.000
Public health	38 (20.4)	13 (7.6)	**0.020**
Total	186 (100)	172 (100)	—

^a^ Pearson's chi‐square test with Bonferroni adjustment. Bolded values represent significance.

To assess the sample's representativeness, we compared age, sex, and institution type (public/private) with 2023 national population data of graduating dental students. While sex distribution showed no significant difference (*p* = 0.321), discrepancies were observed in age (*p* < 0.01) and institution type (*p* < 0.001). However, the observed imbalances necessitate caution when generalizing the results.

## 4 Discussion

Certainly, the most important decision for dental students after graduation is to outline their future career plans [12]. This was the first Brazilian study that investigated the career motivations of final‐year dental students in OP and OM by insights from different public and private Brazilian dental schools.

In the present study, most of the participants were female (75.1%). In recent years, dentistry has changed from being a male‐dominated profession to a female‐dominated one. This is the result of efforts made over the years in some countries, including Brazil, to overcome the sex bias in dentistry, with the aim of achieving sex parity, opportunities, and leadership engagement for women [14, 15]. However, despite increasing female participation, additional efforts should be made, as women are still a minority in leadership positions in the dental workforce [15] and are underrepresented among Brazilian health researchers in terms of scientific productivity [16].

Overall, the students' interest in pursuing a professional career in OM and OP was 17% and 7.5%, respectively. This low interest of dentists in the fields of OM and OP may imply a lack of specialists. This can lead to late or incorrect diagnoses, inadequate treatment of oral diseases, and an overload on health systems, compromising the quality of patient care. This result emphasizes the necessity of efforts to promote the significance of these specialties among dental students. Brazilian specialists in OP and OM have been developing strategies to promote and expand the specialty, with some being members of important societies in these fields [1]. Furthermore, Brazilian scientific production in OP and OM has grown with notable international visibility. Most articles by the Brazilian research productivity grant recipients were published in international journals and involved foreign collaborators [17, 18, 19]. Moreover, Brazilian scientists comprise the editorial board of leading journals in OP and OM [1]. This scenario may explain the influence of professors in choosing the professional career of OM and OP among Brazilian dental students. However, it does not seem to attract more dentists for the practice of OM and OP.

Several factors may influence the decision of future career, such as sex, flexibility of working hours, costs related to education, and family influence [10, 11]. In Brazil, the choice of occupation seems to be influenced by the students' characteristics and their perceptions of the occupation (dos Santos et al., 2022). The present study showed that for OP and OM, vocation and influence from a professor, family member, or colleague are important factors that influence this decision. The literature also supports that mentoring from family, colleagues, and professors are the most important factors in career choice [20]. On the other hand, for restorative dentistry, dental implantology, prosthodontics, and orofacial harmonization, professional distinction and improvement of clinical practice were identified as the main motivations for choosing these fields. A high financial return, a large number of hours in curricular training in these subjects, and an increasing esthetic demand may influence the significant interest in restorative dentistry, dental implantology, prosthodontics, and orofacial harmonization [21]. These findings reinforce the need to expand the fields of professional activity and carry out campaigns to popularize and show to the population the importance of OM and OP. These actions can result in greater demand for OM and OP, with greater financial return and consequent attraction of new professionals for these specialties.

Although no systematic data are available on the remuneration of OM and OP specialists compared with other dental specialties, some evidence suggests that these fields may be less financially attractive. In Brazil, employment outcomes of graduates from OM and OP postgraduate programs are more frequently linked to teaching and research positions, especially in the private sector, rather than to clinical practice with high financial return [22]. Furthermore, qualitative studies indicate that the perception of limited job opportunities, heavy workload, and restricted financial prospects discourages young dentists from pursuing a lifelong career in these specialties [23]. Pinto and Mendes [24] emphasize that innovative business models and broader dissemination of the clinical relevance of OM are necessary to enhance the visibility, sustainability, and compensation of these professional pathways.

A previous study has shown that active members of the American Academy of Oral Medicine have a high career satisfaction working in OM [25]. However, when investigating the interest of young dentists in a lifelong career in OP, most Taiwanese dentists demonstrated a very low interest in this field. The primary factors influencing this decision included learning characteristics, a lack of understanding of the profession, heavy workloads, limited job opportunities, and a poor quality of life [23]. Pinto & Mendes [24] advocate for integrating business modeling principles into current training programs and encouraging experienced practitioners to share their insights. They emphasize that although OM is historically focused on academia, clinicians have the opportunity to enhance patient care and educate both the public and colleagues on the specialty's value. The introduction of innovative technologies such as mucosal medication delivery, telemedicine, stem cell therapy, and advanced treatments for salivary hypofunction requires extensive clinical testing, a role well‐suited for OM dentists. Ultimately, these advancements aim to optimize patient outcomes.

Interest in specializing in OM and OP among dental students varies, with generally low rates reported in various studies. An Indian survey among dental undergraduates found that while 16% of students found OP interesting, a significant portion (64%) focused primarily on passing the subject rather than considering it for postgraduate specialization [26]. Additionally, another study in the US found that the majority of students (64%) perceived the OM specialty as not well‐known, with only 13% considering it familiar. Surprisingly, only 1% of respondents expressed interest in pursuing OM as a future postgraduate specialty [27]. In a study evaluating the interest of dentists in future specialization in China and Japan, the most popular areas were prosthodontics, orthodontics, and oral surgery, with OP and OM not even mentioned [28].

This lack of interest may lead to a shortage of specialists in these critical areas, impacting the quality of care provided to patients. To address this issue, efforts are needed to increase awareness and understanding of OM among students to better meet the healthcare needs of the population, emphasizing the importance of integrating this knowledge into dental school curricula and continuing education programs [27].

Insufficient OM and OP training in the undergraduate dental curriculum may also be related to the lack of engagement in these areas among dental students. In this sense, special care dentists, as related area with OM, reported insufficient knowledge from undergraduate and early postgraduate training, with a lack of enough focus on important topics, leading to disadvantages in professional practice [29].

Some countries have a significant shortage of OP and OM specialists. In the Arab Middle East, OP and OM are not yet recognized as specialties by the Arab Board of Health Specialization. In addition, the scarcity of training programs and career opportunities in these areas may impact the professional career choice of Arab dental students. In Europe, OM is not recognized as a specialty in most countries, and the postgraduate training in this area of dental education is limited. Likewise, employment opportunities are more frequent in academic fields [7].

In a global context, since 2015, the transition from the United Nations' Eight Millennium Development Goals to the 17 Sustainable Development Goals (SDGs) has emphasized health‐related SDGs as critical benchmarks. These goals necessitate continuous monitoring to ensure national and global accountability for the well‐being of the global population. Defined by the World Health Organization as ‘a state of complete physical, mental, and social well‐being and not merely the absence of disease or infirmity,’ health underscores the role of OM specialists. They are recognized as healthcare professionals with a holistic approach to patient care that encompasses both oral and overall health, and also as leaders in oral health care, providing essential technical expertise to the healthcare system [24].

There are 0.48 oral pathologists in median per million of inhabitants. Worldwide, OP training is extremely variable, despite some similarities. Moreover, most OP training is academic, involving master's or PhD degrees. On the other hand, a fellowship or diploma is sufficient for accreditation in some countries [5]. Latin America has a low number of OP and OM specialists for the total population. Currently, there are only 422 OP specialists and 1072 OM specialists for 203 million inhabitants in Brazil [4]. Furthermore, in Brazil, postgraduate training in OP and OM, as well as funding, is concentrated in the Southeast region [22]. Moreover, employment opportunities in these fields in Brazil are more likely to be in the private sector, especially in teaching/research activities [22]. Such outcomes are not yet well understood but appear to contribute to the students' lack of interest in these professional pathways.

This study has several limitations. The use of convenience sampling may lead to selection bias, affecting the generalizability of the findings. While our sample's sex distribution matched the 2023 National Education Census data from INEP, significant discrepancies were observed in both age and institution type distribution when compared to these official national benchmarks. These demographic imbalances, inherent to the convenience sampling approach, may constrain the generalizability of our findings to the broader population of Brazilian dental graduates. The cross‐sectional design captures data at one point in time, limiting the ability to infer causality or track changes and trends over time. The reliance on self‐reported data introduces potential response bias and inaccuracies in participants' reporting. Additionally, the study's limited geographic representation, covering only seven Brazilian states, may restrict the generalizability of the results to the entire country.

The results revealed a surprising and concerning finding: interest in the fields of OP and OM, which are fundamental for diagnosing and treating complex conditions of the oral cavity, was relatively low. This can result in an increase in undiagnosed or inadequately treated cases, affecting the overall health of the population. Additionally, the lack of specialists can overburden other sectors of dentistry, making access to specialized care more difficult and exacerbating inequalities in dental care. This trend raises an alarm about the need for educational and motivational strategies to reverse this situation, ensuring a more balanced and comprehensive future for dentistry. Brazilian groups have been working hard to promote these specialties, but additional efforts should be made to promote these careers among dental students. Furthermore, this study emphasizes the importance of mentoring, the influence of family and professors, and the sense of vocation in the academic pathways for this field.

## Author Contributions

**Hélen Kaline Farias Bezerra:** contributed to study conception and data acquisition; participated in manuscript drafting; approved the final version; accountable for the integrity of the work. **Fabrício Emanuel Soares de Oliveira:** participated in data analysis and interpretation; critically revised the manuscript; approved the final version; accountable for the integrity of the work. **Daniella Reis Barbosa Martelli:** contributed to study design and data interpretation; involved in manuscript drafting; approved the final version; accountable for the integrity of the work. **Renato Assis Machado:** contributed to data acquisition and analysis; participated in manuscript revision; approved the final version; accountable for the integrity of the work. **Zêus Araújo Cunha:** assisted in study design and data interpretation; critically revised the manuscript; approved the final version; accountable for the integrity of the work; **Fábio Ramoa Pires:** contributed to data interpretation; critically revised the manuscript for important intellectual content; approved the final version; accountable for the integrity of the work. **Danyele Cambraia Franco de Souza:** participated in data acquisition and interpretation; involved in manuscript drafting; approved the final version; accountable for the integrity of the work. **Silvia Regina de Almeida Reis:** contributed to data analysis; critically revised the manuscript; approved the final version; accountable for the integrity of the work. **Giovanna Ribeiro Souto:** participated in study conception and manuscript drafting; approved the final version; accountable for the integrity of the work. **Paulo Rogério Ferreti Bonan:** contributed to study design and critical revision of the manuscript; approved the final version; accountable for the integrity of the work. **Danyel Elias da Cruz Perez:** assisted in data interpretation and critical manuscript revision; approved the final version; accountable for the integrity of the work. **Alan Roger dos Santos‐Silva:** contributed to data acquisition and analysis; participated in manuscript drafting; approved the final version; accountable for the integrity of the work. **Luiz Evaristo Ricci Volpato:** participated in study conception and critical revision of the manuscript; approved the final version; accountable for the integrity of the work. **Janete Dias Almeida:** contributed to data interpretation and manuscript drafting; approved the final version; accountable for the integrity of the work. **Hercílio Martelli‐Júnior:** coordinated the study design and conception; contributed to data interpretation and manuscript revision; approved the final version; accountable for the integrity of the work.

## Funding

The authors have nothing to report.

## Conflicts of Interest

The authors declare no conflicts of interest.

## Data Availability

The data that support the findings of this study are available on request from the corresponding author. The data are not publicly available due to privacy or ethical restrictions.
